# Recent trends of refractive surgery rate and detailed analysis of subjects with refractive surgery: The 2008-2015 Korean National Health and Nutrition Examination Survey

**DOI:** 10.1371/journal.pone.0261347

**Published:** 2021-12-23

**Authors:** Yunjin Lee, Ji Soo Kim, Un Chul Park, Juwon Lim

**Affiliations:** 1 Department of Ophthalmology, Seoul National University Hospital, Seoul National University College of Medicine, Seoul, Korea; 2 International Healthcare Center, Seoul National University Hospital, Seoul National University College of Medicine, Seoul, Korea; Universidad de Monterrey Division de Ciencias de la Salud, MEXICO

## Abstract

The present study was conducted to investigate recent trends of refractive surgery rates and analyze subjects undergoing refractive surgery using large-scale population studies over the past 8 years. We used the dataset of the Korean National Health and Nutrition Examination Surveys, a nationwide population-based cross-sectional study which were performed from 2008 to 2015. Of the 21,415 participants aged 20 to 49 years, 1,621 had refractive surgeries. Seventy three percent of them were females and 81% of them were aged under 40 years old. Over the past 8 years, cumulative prevalence of refractive surgery rate increased more than 10%. Although young (< 40 years, odds ratio (OR) 0.31, P<0.001) women (OR 1.86, P<0.001) living in urban areas (OR 0.51, P<0.001) with high educational attainment (OR 2.67, P<0.001) and income levels (OR 3.16, P<0.001) accounted for a high proportion in refractive surgery group through all survey years, subgroup analyses revealed that gaps between genders (ORs 3.8 in 2008–2009, 2.1 in 2010–2012, and 1.5 in 2013–2015), educational level (ORs 3.0, 2.5, and 2.1, respectively), and highest/lowest quartiles of household income (ORs 5.2, 2.6, and 2.4, respectively) were decreasing over time. Overall, our study suggests that refractive surgery has reached an age where the majority accepts it, and indeed more and diverse people are undergoing refractive surgeries.

## Introduction

Myopia and high myopia were estimated to affect 33% (2584 million) and 4.0% (300 million) of the world population respectively in 2020, and these values are anticipated to increase to 52% (4949 million) and 10% (925 million) in 2050 [1]. Particularly, the prevalence of myopia has profoundly increased especially in the younger generation of East Asia [2]. According to data from the Korea National Health and Nutrition Examination Survey (KNHANES, 2008–2009), the prevalence of myopia in children and adolescents in Korea ranges from 50% in children aged 5–11 years to 78.8% in children aged 12–18 years [3]. In 2012, 96.5% and 93.3% of 19-year-old males in urban and rural areas of Korea, respectively, reported to have myopia [4].

Radial keratotomy was introduced as the first refractive surgery in the 1970’s [5]. Following that, photorefractive keratotomy (PRK) was introduced in 1988, and laser in situ keratomileusis (LASIK) in 1990 [5]. Laser epithelial keratomileusis (LASEK) was developed in 1990 to reduce the complications of corneal flap, corneal ectasia, and epithelial ingrowth [6]. Phakic intraocular lenses (IOLs), known as implantable contact lenses (ICLs), are a surgical option for treatment of high myopia [7]. As the safety and effectiveness of refractive surgery have been established along with the increase of prevalence of myopia, the number of people desiring to eliminate the need for eyeglasses or contact lenses and see clearly has also increased from all walks of life.

However, despite huge potential benefits of surgical correction of refractive errors, not everyone undergoes refractive surgery. Furthermore, until now most of published studies focus on surgical techniques, surgical outcomes, and complications [5–8], but seldom include a detailed analysis of the patients who underwent refractive surgeries. The demographics and clinical characteristics of these patients are varied and these data could establish a clinical profile of patients, raise awareness of people undergoing refractive surgeries, and thus suggest direction of advancement that refractive surgeries should consider. Therefore, we evaluated recent trends of refractive surgery rates and detailed analysis of subjects undergoing refractive surgery using large-scale population studies over the past 8 years (KNHANES, 2008–2015).

## Methods

### Data source and study population

The KNHANES is a nationwide cross-sectional study that has been conducted by the Korea Centers for Disease Control and Prevention (KCDC) and the Korean Ministry of Health and Welfare since 1998 [9]. The survey uses a complex, stratified, multistage, probability cluster to obtain a representative sample of the population. The detailed study design of the KNHANES has been published previously [9], and the statistics of the KNHANES are publicly available on the study’s website (http://knhanes.cdc.go.kr).

In this study, the data after 2008 were included because information regarding refractive surgery were collected since 2008. For the same reason, the study population was limited to adults aged 20–49 years to examine trends of refractive surgery. After excluding those who did not complete the ophthalmic examination and those who did not respond to whether having refractive surgery or not, the total number of participants in the analysis was 21,415.

The institutional review board (IRB) of the KCDC reviewed and approved the KNHANES survey annually (IRB approval no.: 2008-04EXP-01-C, 2009-01CON-03-2C, 2010-02CON-21, 2011-02CON-06-C, 2012-01EXP-01-2C, 2013-07CON-03-4C, and 2014-12EXP-03-5C). The Institutional Review Board (IRB) of Seoul National University Hospital approved the present study (IRB identification number: E-2107-145-1236), which was conducted in accordance with the Declaration of Helsinki.

### Health interview and examination

The KNHANES consists of three parts: a health interview, health examination, and nutrition survey. The health interview and examination are conducted by trained staff members in mobile centers that travel to each survey location around the country. Information including age, sex, and sociodemographic factors such as residential area, education level, household monthly income, employment, and types of occupation were obtained through health interviews. Personal indicators regarding subjective body type perception, weight control efforts, mental health (stress level, depressive mood, and suicidal idea), physical activity, smoking, alcohol consumption, marital status, and sleep hours were also investigated.

Residential area was divided into two groups—rural: town [eup], township [myeon], or neighborhood [dong] and urban: city [si], county [gun], and district [gu]. Education level was grouped into two categories: college or higher (≥13 years), and high school or less (≤12 years). We divided study participants into four groups according to quartiles of household income (1Q-4Q; 1Q, lowest quartile; 4Q, highest quartile). Groups of working individuals were divided into two groups: white collar workers and blue collar workers. White collar workers refer to general office workers and management. Blue collar workers refer to those who perform manual labor.

The participants also underwent ophthalmologic questionnaires which included history of refractive surgery. However, specific method of the surgery (eg, LASIK, LASEK, PRK, or phakic IOLs implantation) and date of the surgery were not inquired.

### Ophthalmic examination and variable definition

Ophthalmic examinations were conducted under the supervision of the Epidemiologic Survey Committee of the Korean Ophthalmological Society [10]. Assessment of visual acuity (VA) was done as follows; initially, uncorrected VA, corrected VA with the participant’s own glasses or contact lens, or VA after refractive surgery were measured at a distance of 4 m using an international standard vision chart based on the Snellen Scale (Jin’s vision chart, Seoul, Korea). Participant VA was measured in each eye, right side followed by left side. Each participant was asked to read numbers in the 0.2 line of the VA chart and to proceed to the next line if he or she correctly read at least three of the five letters. Participant VA was defined as the line with the smallest numbers in which the participant accurately read more than three characters. For those participants who presented with VA score lower than 0.8, corrected VA was measured using autorefraction. Automated refraction (AR) was performed without cycloplegia using an autorefractive keratometer (KR8800; Topcon, Tokyo, Japan), followed by VA retesting using a pinhole in patients with Snellen VA lower than 0.8. Refraction measurements were converted into spherical equivalents (SE), calculated as the spherical value plus half of the cylindrical value.

### Statistical analysis

As data from KNHANES were derived from stratified and multistage clustered probability sampling methods to represent the entire Korean population, population weightings were also applied in the analyses.

Pearson’s Chi-square test was used for statistical comparison among subgroups for estimated weighted prevalence. Crude prevalence ratios were obtained using a separate logistic regression model for each of the independent variables. All variables that were in simple logistic models were included in initial multivariate modeling. The categorical variables of the study participants are presented as 95% confidential intervals (CI) or age-adjusted proportion.

Comparisons of the characteristics across survey periods were performed using analysis of variance or Chi-square test, as appropriate, and Chi-square linear trend test was also used. To adjust the changes in age structure in study population from each survey, age-adjusted prevalence was calculated using a direct standardization method based on 2010 Korean Census.

Significant levels were set at a two-tailed P value <0.05. All analyses were conducted using STATA V.14.2 (Stata Corp, College Station, Texas, USA, 2013).

## Results

### General characteristics of participants

We analyzed the general characteristics of the study participants (**Table 1**). Of the 21,415 participants, 1,621 underwent refractive surgeries. Among them, 26.2% (425) were males and 73.8% (1,196) were females. The participants who did not have refractive surgery consisted of 52.6% (8,665) of males and 47.4% (11,129) females. When divided into 3 groups (20–29, 30–39, and 40–49 years old) according to age, the proportions of the non-surgery group were similar (29.1%, 33.3%, and 37.6%, respectively), however in the surgery group, 81% of the participants were aged under 40 years old and only 19% were over 40 years old. In distribution of residential areas, about 85.1% of those without surgery and 91.5% of those with surgery were identified as urban residents. In terms of education level, 45.3% of the non-surgery group and 71.7% of the surgery group had a college or higher academic background. In the aspect of household monthly income, the non-surgery group showed a relatively similar proportion (27.1%, 26.4%, 23.0%, and 23.5%, 1Q-4Q, respectively), however in the surgery group, the rate corresponding to 4Q was 34.8% while 1Q was only 14.8%.

**Table 1:** Baseline characteristics of the study population.

	No surgeries		Refractive surgeries		P-value
	N	Weight % (95% CI)	N	Weight % (95% CI)	
	19794	92.6(92.2–93.1)	1621	7.4(6.9–7.8)	
Gender					<0.001
Men	8665	52.6(51.9–53.3)	425	26.2(24.1–28.4)	
Women	11129	47.4(46.7–48.1)	1196	73.8(71.6–75.9)	
Age groups					<0.001
20–29	4540	29.1(28.1–30.1)	524	32.3(30.1–34.6)	
30–39	7257	33.3(32.3–34.3)	789	48.7(46.2–51.1)	
40–49	7997	37.6(36.6–38.7)	308	19.0(17.2–21.0)	
Residual area					<0.001
Urban	16803	85.1(83.1–86.9)	1483	91.5(90.0–92.8)	
Rural	2991	14.9(13.1–16.9)	138	8.5(7.2–10.0)	
Education level					<0.001
≤High school	10099	54.7(53.6–55.8)	440	28.3(26.1–30.6)	
≥College	8995	45.3(44.2–46.4)	1114	71.7(69.4–73.9)	
House monthly income					<0.001
1Q	1486	27.1(25.4–28.9)	56	14.8(12.6–17.4)	
2Q	4887	26.4(24.7–28.1)	289	25.0(22.2–28.0)	
3Q	6777	23.0(21.5–24.6)	491	25.4(22.5–28.4)	
4Q(high)	6467	23.5(21.7–25.3)	770	34.8(31.7–38.1)	
Job style					<0.001
White collar	9330	48.7(47.8–49.6)	879	56.8(54.3–59.2)	
Blue collar	3664	21.0(20.1–21.8)	95	6.1(5.0–7.4)	
None	6031	30.3(29.5–31.1)	574	37.1(34.7–39.5)	
Marital status					<0.001
Single	5187	32.5(31.3–33.8)	608	37.7(35.3–40.1)	
Married	14582	67.5(66.2–68.7)	1006	62.3(59.9–64.7)	
Smoking					<0.001
Never	11139	52.8(51.9–53.6)	1147	72.5(70.2–74.6)	
Past	3047	17.7(17.0–18.4)	202	12.8(11.2–14.5)	
Current	5095	29.6(28.7–30.4)	234	14.8(13.1–16.6)	
Alcohol(drink/day)					<0.001
<1	10958	66.9(66.0–67.9)	1056	78.6(76.3–80.7)	
≥1	5005	33.1(32.1–34.0)	288	21.4(19.3–23.7)	
Exercise, walk(min/day)					<0.001
≥30	8957	46.7(45.8–47.6)	819	52.7(50.2–55.1)	
<30	10138	53.3(52.4–54.2)	736	47.3(44.9–49.8)	
Sleep(hour/day)					0.115
<6	1875	9.7(9.2–10.2)	121	7.7(6.4–9.1)	
6–8	16011	83.0(82.4–83.7)	1334	84.5(82.6–86.2)	
≥8	1385	7.3(6.8–7.8)	124	7.9(6.6–9.3)	
Stress					0.452
None or Low	13241	69.6(68.7–70.4)	1110	70.1(67.8–72.3)	
High or Moderate	6046	30.4(29.6–31.3)	473	29.9(27.7–32.2)	
Depressive mood					0.230
No	15289	89.0(88.4–89.6)	1171	89.8(88.0–91.3)	
Yes	2005	11.0(10.4–11.6)	133	10.2(8.7–12.0)	
Suicide idea					0.010
No	15416	89.8(89.2–90.4)	1193	91.5(89.8–92.9)	
Yes	1877	10.2(9.6–10.8)	111	8.5(7.1–10.2)	
Subjective body type perception					<0.001
Normal or thin	10301	54.3(53.3–55.2)	963	60.8(58.4–63.2)	
Obese	8987	45.7(44.8–46.7)	620	39.2(36.8–41.6)	
Weight control efforts					<0.001
No	6236	32.7(31.8–33.5)	452	28.6(26.4–30.8)	
Yes	13048	67.3(66.5–68.2)	1131	71.4(69.2–73.6)	
Visual activity, right					<0.001
20/20	11896	59.9(59.0–60.8)	1202	74.2(72.0–76.2)	
20/25	4315	21.6(20.8–22.3)	296	18.3(16.5–20.2)	
≤20/32	3583	18.5(17.8–19.3)	123	7.6(6.4–9.0)	
Spherical equivalent					<0.001
<-0.5D	7830	39.3(38.4–40.2)	956	59.0(56.6–61.3)	
-3D and ≤-0.5D	7364	37.1(36.3–38.0)	630	38.9(36.5–41.3)	
-6D and ≤-3D	3359	17.3(16.6–17.9)	31	1.9(1.3–2.7)	
≥-6D	1241	6.3(5.9–6.7)	4	0.2(0.1–0.7)	

Persons aged 20–49 years, KNHANES 2008–2015

Abbreviations: CI = confidence interval; 1Q-4Q; 1Q = lowest quartile, 4Q = highest quartile, D = diopters

Chi-square test of difference at each level of sex, age, residual area, education level, house monthly income, job style, marital status, smoking, alcohol, exercise, sleep hour, stress, depressive mood, suicidal idea, subjective body type perception, and weight control efforts

Significant levels were set at a two-tailed P value <0.05.

### Sociodemographic and individual indicators of refractive surgery group

We evaluated sociodemographic and personal indicators of the refractive surgery group compared to that of the non-surgery group (**Table 2**). In an univariate analysis, the group with refractive surgery was characterized in terms of higher proportion of women (odds ratio (OR) 1.86, P<0.001), younger age (< 40 years, OR 0.31, P<0.001), high education background (OR 2.67, P<0.001), high income level (OR 3.16, P<0.001), and lower proportion of rural residents (OR 0.51, P<0.001) and blue collar jobs (OR 0.30, P<0.001). Single status, never smoking, regular exercise, subjective perception of normal to slim body shape, and weight control efforts (all, OR 1, P<0.001) were the individual characteristics of the surgery group distinguished from the non-surgery group. In a fully adjusted multivariate analysis, the statistically significant adjusted OR (P<0.05) for refractive surgery were age, sex, residential area, educational level, household income, job style, marriage, smoking, and subjective body type perception (**Table 2**).

**Table 2:** Adjusted odds ratio of refractory surgeries among adult in South Korean population.

	Univariate analysis		Multivariate analysis	
	OR(95% CI)	P-value	aOR(95% CI)	P-value
Gender				
Men	1		1	
Women	1.86(1.62–2.12)	<0.001	1.95(1.62–2.33)	<0.001
Age groups				
20–29	1		1	
30–39	0.99(0.84–1.15)	0.862	1.01(0.83–1.23)	0.944
40–49	0.31(0.26–0.38)	<0.001	0.39(0.31–0.51)	<0.001
Residual area				
Urban	1		1	
Rural	0.51(0.39–0.67)	<0.001	0.72(0.57–0.89)	0.003
Education level				
≤High school	1		1	
≥College	2.67(2.33–3.07)	<0.001	1.87(1.61–2.17)	<0.001
House monthly income				
1Q	1		1	
2Q	1.57(1.17–2.10)	<0.001	1.35(1.08–1.69)	0.267
3Q	1.92(1.45–2.55)	<0.001	1.78(1.45–2.19)	0.065
4Q(high)	3.16(2.39–4.17)	<0.001	2.70(2.21–3.30)	0.006
Job style				
White color	1		1	
Blue color	0.30(0.23–0.39)	<0.001	0.70(0.54–0.91)	0.008
None	0.99(0.85–1.14)	0.843	1.02(0.88–1.18)	0.810
Marital status				
Single	1		1	
Married	0.59(0.51–0.68)	<0.001	0.77(0.64–0.94)	0.009
Smoking				
Never	1		1	
Past	0.67(0.56–0.81)	<0.001	0.94(0.76–1.16)	0.552
Current	0.53(0.45–0.64)	<0.001	0.78(0.63–0.96)	0.020
Alcohol(drink/day)				
<1	1		1	
≥1	0.67(0.57–0.80)	<0.001	0.92(0.77–1.09)	0.343
Exercise, walk(min/day)				
≥30	1		1	
<30	0.75(0.66–0.86)	<0.001	0.94(0.82–1.07)	0.336
Sleep(hour/day)				
<6	1		1	
6–8	1.25(0.98–1.59)	0.067	1.07(0.84–1.37)	0.586
≥8	1.38(1.00–1.89)	0.049	0.92(0.65–1.29)	0.614
Stress				
None or Low	1		1	
High or Moderate	0.95(0.83–1.09)	0.452	0.92(0.79–1.07)	0.264
Depressive mood				
No	1		1	
Yes	0.88(0.70–1.11)	0.296	0.94(0.74–1.18)	0.586
Suicide idea				
No	1		1	
Yes	0.71(0.55–0.92)	0.011	0.91(0.71–1.16)	0.447
Subjective body type perception				
Normal or thin	1		1	
Obese	0.74(0.65–0.84)	<0.001	0.81(0.71–0.93)	0.002
Weight control efforts				
No	1		1	
Yes	1.26(1.10–1.46)	0.001	1.14(0.98–1.32)	0.091

Persons aged 20–49 years, KNHANES 2008–2015

Abbreviations: OR = Odds ratio; aOR = adjusted odds ratio; CI = confidence interval; 1Q-4Q; 1Q = lowest quartile; 4Q = highest quartile

Chi-square test of difference at each level of sex, age, residual area, education level, house monthly income, job style, marital status, smoking, alcohol, exercise, sleep hour, stress, depressive mood, suicidal idea, subjective body type perception, and weight control efforts

Significant levels were set at P value <0.05 for univariate analysis and multivariate analysis.

Therefore, it can be considered that representative characteristics of the refractive surgery group are being young, highly educated, unmarried women who live in the urban and have high-income white collar jobs.

### Trends of refractive surgery rate among aged 20–49 years in 2008–2015 in Korea

**Fig 1:**
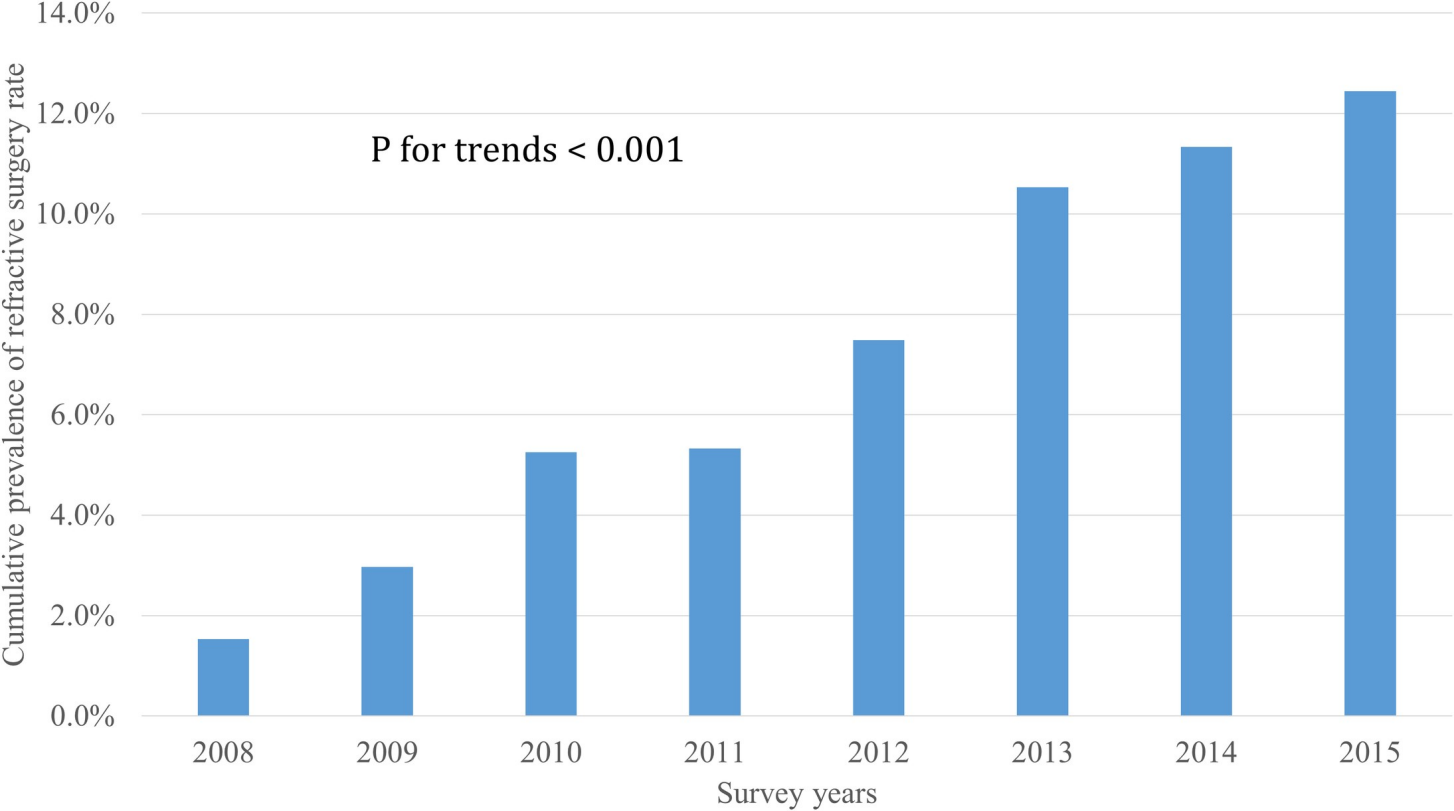
Cumulative prevalence of refractive surgery rate by survey years, KNHANES 2008–2015. The cumulative proportion of participants who underwent refractive surgery showed an increasing trend. P for trends <0.001.

Over the past 8 years, the cumulative proportion of participants who underwent refractive surgery showed an increasing trend. Specifically, 1.5% of study participants in 2008, 3.0% in 2009, 5.3% in 2010 and 2011, 7.5% in 2012, 10.5% in 2013, 11.3% in 2014, and 12.4% in 2015 (**Fig 1**) responded that they had undergone refractive surgery.

**Fig 2:**
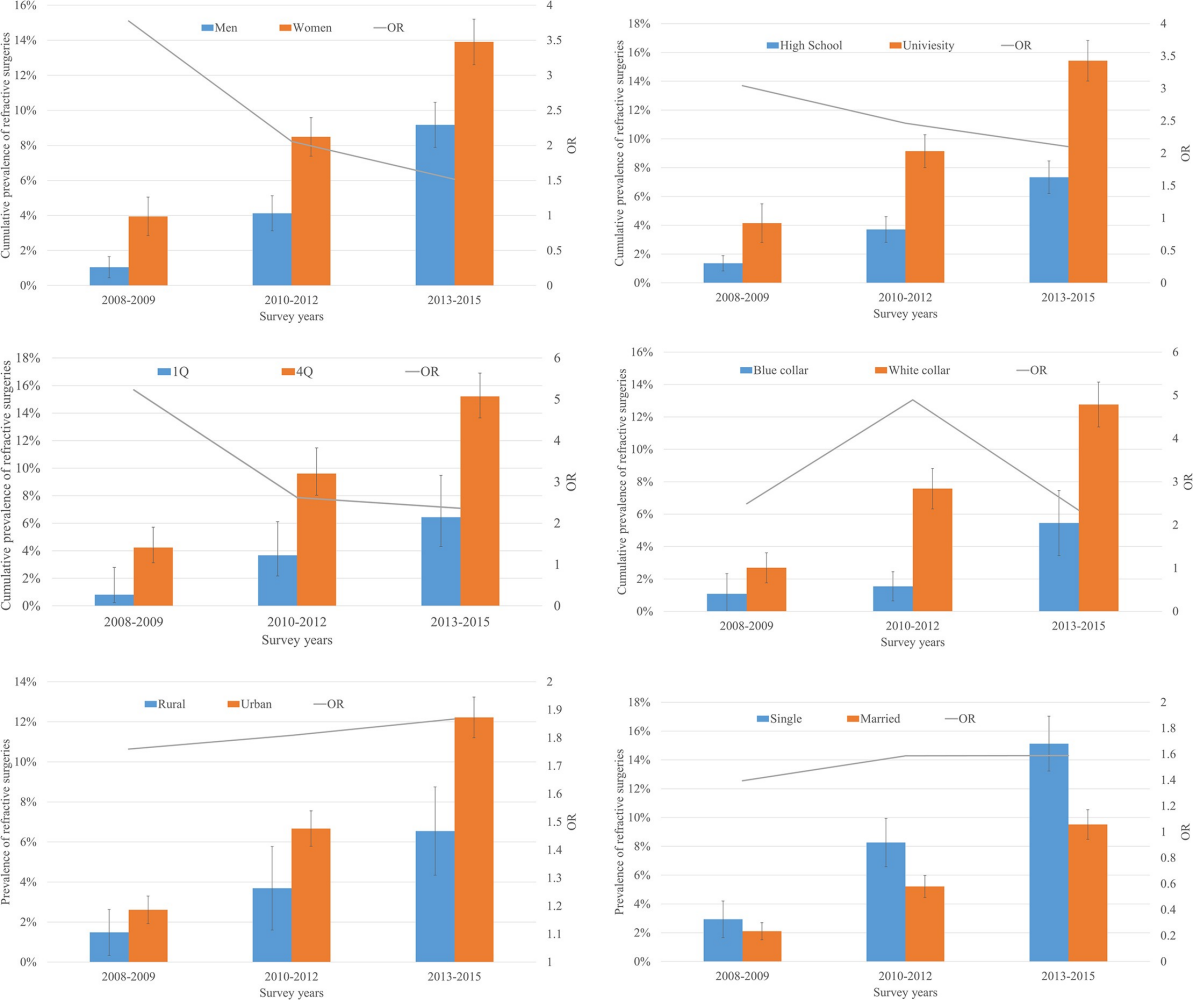
Comparison of prevalence of refractive surgeries divided by sub-groups. Cumulative prevalence of refractive surgeries showed that gaps between (A) genders (men/women), (B) educational level attainment (high school or less/college or higher), (C) household monthly income level (lowest/highest quartile), and (D) job style (blue collar/white collar) were decreasing over the time. When it comes to (E) residential areas (rural/urban) and (F) marital status, the gaps were widening over the time, but the differences of the gaps were decreasing. Abbreviations: OR = Odds ratio; 1Q = lowest quartile; 4Q = highest quartile.

In a time trend analysis, as for the rate of men and women who underwent refractive surgery, women showed higher rate throughout all survey years (Fig 2A). Whether there was a change within the refractive surgery group over time, subgroup analyses were performed. In a subgroup analysis divided by gender (men/women), the gaps between the two groups were gradually decreasing. Specifically, the odds ratios (ORs) were 3.8 in 2008–2009, 2.1 in 2010–2012, and 1.5 in 2013–2015 (Fig 2A). As for the educational level (high school/university), the gaps between the two groups also showed declining trend. In more detail, the ORs were 3.0, 2.5, and 2.1, respectively (Fig 2B). In comparison by household income (1Q/4Q), the ORs were 5.2, 2.6, and 2.4, respectively (Fig 2C), which meant the gaps were reducing. Pertaining to the subgroup analyses of residential area (rural/urban) and marital status (single/married), although the gaps were widening over the time, the differences of the gaps were decreasing (Fig 2E and 2F).

### Visual acuity and spherical equivalents of refractive surgery group

The SE values in both eyes were highly correlated in Pearson’s correlation analysis (r = 0.881, P <0.01). Therefore, in this study, we used the right eye for analysis.

About 92.5% (1,498) of participants maintained VA over 20/25 after refractive surgeries, although SE less than -0.5 diopter were 59% (**Table 1**). Among participants with refractive surgery, 2.2% (35) answered wearing glasses or contact lenses after the surgical correction of refractive errors. However, specific reasons of wearing glasses or contact lenses were not inspected.

## Discussions

According to a previous study based on the data from KNHNES 2008–2012, the prevalence of myopia children aged 5–18 years in Korea was 64.6% [11]. Among them, there might be those who prefer to wear glasses or contact lenses or could not consider refractive surgery due to accompanying ophthalmic or other systemic diseases. Even so, the majority are potential candidates for refractive surgeries. From the results of our study, it was revealed that cumulative prevalence of refractive surgery rate increased more than 10% in a sample survey over the past 8 years. Although these increasing rates cannot be guaranteed, if myopic patients receive refractive surgery at this current rate, it seemed necessary to evaluate and recognize characteristics of patients undergoing refractive surgery and consider direction of future research and development.

Compared to those without surgery, people with refractive surgery mainly composed of women residing in urban area with high level education, employed in general office works or management. However, in a time trend analysis, it was found that the gap which made categorization of certain classes were reducing. These phenomena could be interpreted as refractive surgery expanding from young, well-educated, high-income, urban-dwelling women representing early adopters, to a broad, diverse, and unspecified population. Just as the hierarchical diffusion of new cultures and technologies [12, 13], the acceptance of refractive surgery has reached a transitional period from early adopters and friendly early majorities to later practical majorities. Particularly, the data on gaps according to monthly income levels reduced from 6 to 3 times and to 2 times could be decisive evidence to say that refractive surgery has reached a time to be accepted by the pragmatic majorities.

It has been revealed that individuals in a variety of occupations had undergone refractive surgeries. They not only work in the office but may also work in diverse work spaces including dusty, dry, or humid outdoor, or in specific occupational environments with exposure to high altitude, high acceleration, low oxygen, or strong ultraviolet rays. Since most of them are under the age of 40, which is a period of active social life, socially and institutionally appropriate eye protection should be prepared in place. Even with mild trauma, people who have undergone LASIK surgery can suffer from flap-related complications. Also, further studies to determine the effect of particular environments to the long-term outcome of refractive surgeries are required. In addition, as it is a surgery that people receive in their 20s or 30s when the occupation has not yet been decided or the field of works may change in the future, current situations might not be the same in the future even if it were applicable at the time of surgery. Therefore, surgeons should keep in mind characteristics of recipients and fully explain the benefits and risks of surgery before choosing surgical options and also after the surgeries. Although the safety of refractive surgery has been proven for about 20–30 years, since remaining life expectancy is quite long and the possibility of change is quite high for young receivers, it is required to continuously monitor the change of ocular biometry and ophthalmic condition, side effects, and complications of the refractive surgeries in relation to recipient’s specific circumstances.

There are several limitations in this presented study. First, the KNHANES is a cross-sectional study, thus the correlations cannot be interpreted as causal relationships. Second, since the records of refractive surgery were based on self-reported questionnaires, it may not be as accurate as medical records. For the same reason, specific types of surgery, timing of the surgery, and re-operation cannot be determined. Third, cautions are needed in interpretation of the data since these surveys did not indicate the number and rate of refractive surgeries performed in the corresponding year, but refer to the cumulative numbers of surgeries in the age group in the survey years. Fourth, as KNHANES is an annual rolling sampling survey, it may represent overall national characteristics, but cannot reflect each individual characteristics and have no continuity. Nevertheless, our study has its strength in that this is the first study which investigated detailed characteristics of participants with refractive surgeries in Korea. In particular, the observation of changes by analyzing the time period is important in that it can suggest the directions society should pursue and refractive surgery should develop. In this study, we tried not to introduce bias and to preserve the representativeness of the population methodologically.

## Conclusion

The recent trends of refractive surgeries are rapidly evolving and expanding focusing on the convenience of those who consider surgery as well as the therapeutic aspect to correct refractive errors. Although young women living in urban area with high educational background and income level took major proportion of recipients, it has been confirmed that refractive surgeries are being accepted by the growing and diverse majority in this study. Therefore, further research regarding surgical methods and prognosis with respect to various individual circumstances as well as continuous follow-up observations are required.

## Supporting information

S1 File (ZIP)

S1 Data Code book for STATA. (DOCX)

S2 Data (DTA)
