# Supplementary material for: Recent trends of refractive surgery rate and detailed analysis of subjects with refractive surgery: The 2008-2015 Korean National Health and Nutrition Examination Survey
Source: PLoS One. 2021 Dec 23;16(12):e0261347. doi: 10.1371/journal.pone.0261347 (PMC8699644; doi:10.1371/journal.pone.0261347)
Supplement: S1 File — (ZIP) [file pone.0261347.s001.zip › CODE BOOK for STATA 2.docx]

CODE BOOK for STATA

Variables and Labels

| Name of Variables | Labels |
| --- | --- |
| year | Survey year |
| age | Age(year) |
| sex | Gender |
| town_t | Residual area |
| ainc | Home income per month |
| cfam | Numbers of family |
| edu | Education level |
| ec_occp | Job style |
| marri_1 | Marital status |
| bs1_1, bs3_1 | Smoking |
| bd1,bd1_1,bd2_1 | Alcohol consumption |
| be3_33 | Physical activity |
| bp8 | Sleep hours |
| bp1 | stress |
| bp5 | Depressive mood |
| bp6_10 | Suicide idea |
| bo1 | Subjective body type perception |
| bo2_1 | Weight control effort |
| eyeop | history of refractive surgery |
| e_cr_1 | Visual activity, right |
| ser | Spherical equivalent |
